# Hydrophobin Film Structure for HFBI and HFBII and Mechanism for Accelerated Film Formation

**DOI:** 10.1371/journal.pcbi.1003745

**Published:** 2014-07-31

**Authors:** Aniket Magarkar, Nawel Mele, Noha Abdel-Rahman, Sarah Butcher, Mika Torkkeli, Ritva Serimaa, Arja Paananen, Markus Linder, Alex Bunker

**Affiliations:** 1 Centre for Drug Research, Faculty of Pharmacy, University of Helsinki, Helsinki, Finland; 2 Institute of Biotechnology, University of Helsinki, Helsinki, Finland; 3 Department of Physics, University of Helsinki, Helsinki, Finland; 4 VTT Technical Research Centre of Finland, Espoo, Finland; 5 School of Chemical Technology, Aalto University, Espoo, Finland; Institut Pasteur, France

## Abstract

Hydrophobins represent an important group of proteins from both a biological and nanotechnological standpoint. They are the means through which filamentous fungi affect their environment to promote growth, and their properties at interfaces have resulted in numerous applications. In our study we have combined protein docking, molecular dynamics simulation, and electron cryo-microscopy to gain atomistic level insight into the surface structure of films composed of two class II hydrophobins: HFBI and HFBII produced by *Trichoderma reesei*. Together our results suggest a unit cell composed of six proteins; however, our computational results suggest P6 symmetry, while our experimental results show P3 symmetry with a unit cell size of 56 Å. Our computational results indicate the possibility of an alternate ordering with a three protein unit cell with P3 symmetry and a smaller unit cell size, and we have used a Monte Carlo simulation of a spin model representing the hydrophobin film to show how this alternate metastable structure may play a role in increasing the rate of surface coverage by hydrophobin films, possibly indicating a mechanism of more general significance to both biology and nanotechnology.

## Author Summary

Filamentous fungi release a specific type of protein, belonging to a protein family known as “hydrophobins” into their environment to control interfaces in a fashion that promotes growth. Such protein coatings are the mechanism that allows for the mycelia to grow out of the water and into the air. When these hydrophobins form films at the air-water interface and on the surface of solid objects immersed in water, they impart properties to those surfaces that has led to their use in a wide range of industrial applications. Of particular interest is the properties they impart to air liquid interfaces, and as a mechanism to bring protective materials to coat nanoparticles in nanotechnology applications. A more detailed knowledge of the structure of these surfaces will allow for augmentation of their function that is possible through genetic engineering of the hydrophobins themselves. In this study we have combined computational and experimental methods to develop atomistic level insight into the structure of this surface for two important hydrophobins: HFBI and HFBII of *Trichoderma reesei*. In addition to insight into the surface structure, we have uncovered an intriguing possible new mechanism for film formation, which may explain some of the striking properties of hydrophobin films, and could be extended to a more general mechanism.

## Introduction

Hydrophobins are a group of proteins produced by filamentous fungi [1]–[4]. They assemble at surfaces, and perform their function through the alteration of these surfaces. Functions performed by hydrophobins through this mechanism include the lowering of the surface tension of water, and adding a hydrophobic coating to the mycelia to allow for aerial growth [5], adhesion to surfaces [6], and coating of a variety of fungal structures [7], [8]. Hydrophobins can be seen as a mechanism through which the fungi fine tune the properties of interfaces in their environment, resulting in their invasive and adaptive behaviour. When hydrophobins locate to surfaces they are known to form assemblies with long range order [9]–[11]. In addition, the presence of hydrophobin coatings on interfaces is known to affect the properties of the interfaces in a highly specific fashion, beyond merely decreasing surface tension. In particular it has been observed that hydrophobin films at the air-water interface have an elasticity orders of magnitude higher than that observed for other surfactants [12]. Surface-adhered hydrophobin films also display additional unique characteristics [13].

Hydrophobins are divided into two classes, class I and class II hydrophobins. For class I, highly characteristic structures, named rodlets [14], are formed. An example of a class I hydrophobin is the hydrophobin EAS [15], [16]. Class II hydrophobins do not show rodlet structures, instead, they are amphiphilic and form 2D crystalline films on the air-water interface, as confirmed through grazing incidence small angle X-ray scattering (GISAXS) [9]–[11]. In both cases, the unique macroscopic properties observed in hydrophobin films will arise from cooperative effects in the stabilisation of the film that result from the interactions between the interlocking proteins in the structured surface network that they form.

In addition to their role as an adaptive strategy of the fungi that produce them, the interfacial assemblies of hydrophobins have led to numerous industrial applications [17]. Examples include foams [18], protein immobilisation [19], emulsification [20], and dispersion of insoluble compounds [21], [22]. Most of these applications rely on the unique properties of the interfacial films that hydrophobins form. It is particularly noteworthy that the above mentioned exceptional properties can be found on a wide variety of different interfaces, including liquid-liquid and gas-liquid interfaces.

Two hydrophobin proteins, that are of particular interest in relation to their possible industrial applications, are produced by *Trichoderma reesei*, known as HFBI and HFBII, that belong to the class II family of hydrophobins. Their property of forming amphiphilic 2D crystalline structures at the air-water interface, rather than rodlet structures, allows for these structures to be transferred to hydrophobic surfaces where they play a role in bringing macromolecules, to which the proteins are attached, to these surfaces, for example hydrophobic nanoparticles [22]. Some progress has been made in the determination of the structure and mechanisms involved in the formation of HFBI and HFBII films. The first high resolution structure of a hydrophobin, determined through x-ray crystallography, was obtained for HFBII of *T. reesei* [23]. It was found that the protein structure is crosslinked by disulphide bridges and has a diameter of approximately 2 nm. A clearly distinguishable patch on one side of the protein consists of only aliphatic amino acid side chains. This results in an amphiphilic structure with an exposed and flat hydrophobic face. A high resolution crystal structure has now also been determined for HFBI [24], showing a similar structure. Further insight into the structure of films of HFBI and HFBII has been obtained from GISAXS [9]–[11] and Langmuir-Blodget films [9], indicating a triangular lattice symmetry [9] with unit cell sizes of 55 and 56 Å respectively.

We have combined cryo-EM measurement, protein-protein docking and molecular dynamics simulation, to obtain a detailed atomic resolution picture of the assembled structure of hydrophobin films at the air-water interface. We have studied the structures of HFBI and HFBII of *T. reesei* for which high resolution structures of both are available [23]–[27]. Our protein-protein docking results indicate a unit cell composed of six proteins with a structure very close to a P6 2D point group symmetry class [28]. Electron cryomicroscopy results, however, indicate a structure with only P3 symmetry - possibly a structure connecting two air-water interfaces in a thin film. In addition, through the protein-protein docking results we have found a possible metastable structure with P3 symmetry and a smaller lattice size, and have used a Monte Carlo simulation of a simplified model of the surface to demonstrate the role this alternate possible ordering could have in the formation of the surface structure.

## Results

### Protein-protein docking results give surface structure with near P6 symmetry

**Figure 1:**
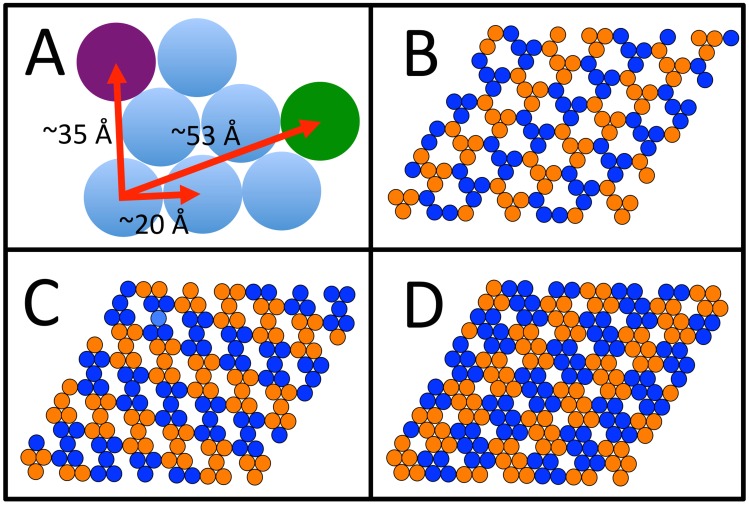
Graphical demonstration of our reasoning regarding possible structures. a) given that hydrophobin protein on hydrophobic surface has a diameter of ∼20 Å, for proteins to be in contact two possible lattice vectors with triangular symmetry can be seen, length ∼35 Å and ∼53 Å. Since experimental results show for HFBI and HFBII the lattice vectors are ∼54 Å and ∼55 Å respectively, this precludes the first lattice vector (∼35 Å). If we constrain the proteins to be in contact with a neighbor, then there exist only three possible structures that will possess this symmetry, b) c) and d).

As shown in Fig. 1a the experimentally observed unit cell size for the 2-D crystal structure of the hydrophobins matches one of the lattice vectors of a close packed (hexagonal) arrangement of the proteins, *i.e.* 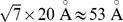. This resulting unit cell may in principle host seven protein molecules, however, this surface would be seen by AFM as a uniform sheet, and published results show this not to be the case [29], [30]. By removing one or more proteins per unit cell we obtain four protein “tetramer” (Fig. 1b), five protein “pentamer” (Fig. 1c) and six protein “hexamer” (Fig. 1d) structures. These represent the only possible structures that are interconnected throughout the 2-D surface. In all cases, they are composed of *trimer* units, by which we mean a set of three mutually interacting proteins. The tetramer structure requires that the proteins have three fold symmetry, which our hydrophobin proteins clearly do not, thus we shall consider only the pentamer and hexamer structures for protein docking.

**Figure 2:**
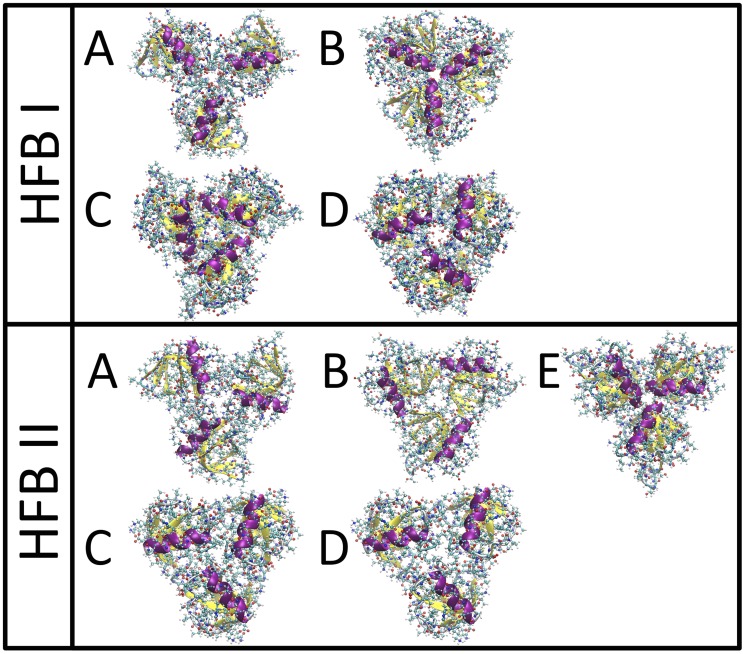
Docking results for HFBI and HFBII fitting three protein unit (trimer). All structures within the top 1% scored are included; four different structures were found (A–D) for HFB I and 5 different structures were found (A–E), for HFB II.

Our first step was to perform protein-protein docking calculations of three proteins, “trimers”, for both HFBI and HFBII. The orientation of the proteins is further constrained so that the main hydrophobic surface orients to the air-water interface. We used protein-protein docking software following the protocol described in the methods section. Selecting all structures with scores in the top 1%, we found four trimer structures for HFBI and five structures for HFBII. These structures are all shown in Fig. 2. Structures C and D for HFBII (see Fig. 2) are extremely similar, with a very small RMSD between them, thus it can be assumed that these are the same structure.

**Figure 3:**
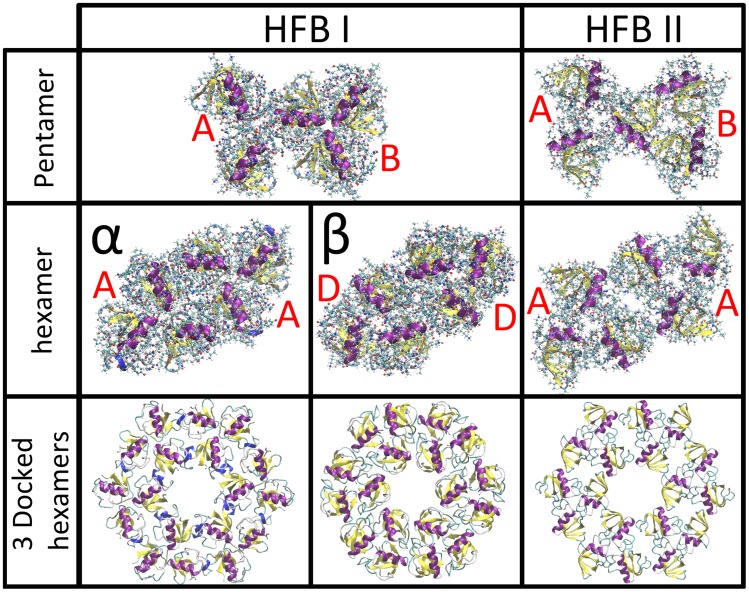
All docking results for possible unit cells composed of previously determined trimers. For both HFBI and HFBII pentamers composed of trimers A and B, in each case the two trimers sharing a protein, could be constructed. These pentamers could not be docked to themselves thus they can not form a unit cell. For both HFBI and HFBII, hexamers could be constructed from two identical trimers, for HFBI from both trimer A and D (see figure 2), to form structures *α* and *β*, and for HFBII, from trimer A. In all three of these cases the hexamers could be successfully docked to themselves, as can be seen from the “18mers”, composed of three hexamers docked in a ring.

For both HFBI and HFBII, it is possible to construct the pentamer structure by combining the trimers A and B (see Fig. 2) and the resulting structures are shown in Fig. 3. In order for this to be the unit cell of the surface layer, the resulting pentamers must be capable of “docking” to each other, in the arrangement shown in Fig. 1 (c). We attempted to perform protein-protein docking of this structure with itself, but this was unsuccessful. Thus we are able to rule out this structure.

**Figure 4:**
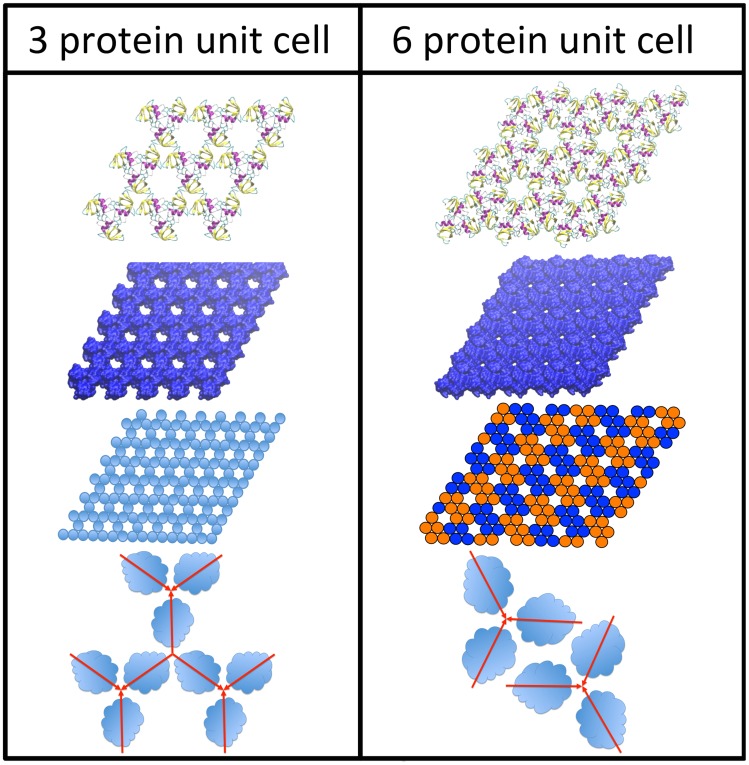
Comparison of the 3 protein and 6 protein unit cell structures for HFBII, the 6 protein unit cell is the better fit to the experimentally observed lattice parameter and provides greater coverage of the surface. Schematics of the P3 and P6 unit cells of the respective structures are shown at the bottom.

We are thus left with the six protein unit cell structure shown in Fig. 1 (d). A unit cell of this structure would be composed of two “docked” trimers, either different or identical. To determine possible combinations, docking was performed for all possible pairs of trimers. In no case did any trimer dock to a trimer different from itself. For HFBI we found two solutions: both trimer A and D were able to successfully dock with themselves, for two structures, that we will name structure *α* and structure *β*. For HFBII only trimer A was found to dock with itself. These results are also shown in Fig. 3. Once again, in order for this structure to be a possible unit cell, it must be able to dock with itself, and in this case our docking of three of these structures was successful, as shown in Fig. 3. It can be seen that the ring structure, found in Fig. 3, is an element of the surface structure composed of the six protein unit cells, seen in Fig. 1 (d), and shown in the six protein structure in Fig. 4.

Now that we have determined that the structure is composed of two identical trimers in the unit cell we may refine the results by symmetry arguments. First, all the docked trimers are within the accuracy range of structures with trigonal symmetry, *i.e.* a 3-fold symmetry axis at the point where the three proteins contact. Further, the two docked trimers are at the same height and are seen to be oriented so that there is a two-fold rotation axis at the midpoint between the trimer axes. Docking the resulting hexamers to themselves was successful (Fig. 3), resulting in an arrangement with P6 symmetry [28] in the unit cell (see six protein structure in Fig. 4).

**Figure 5:**
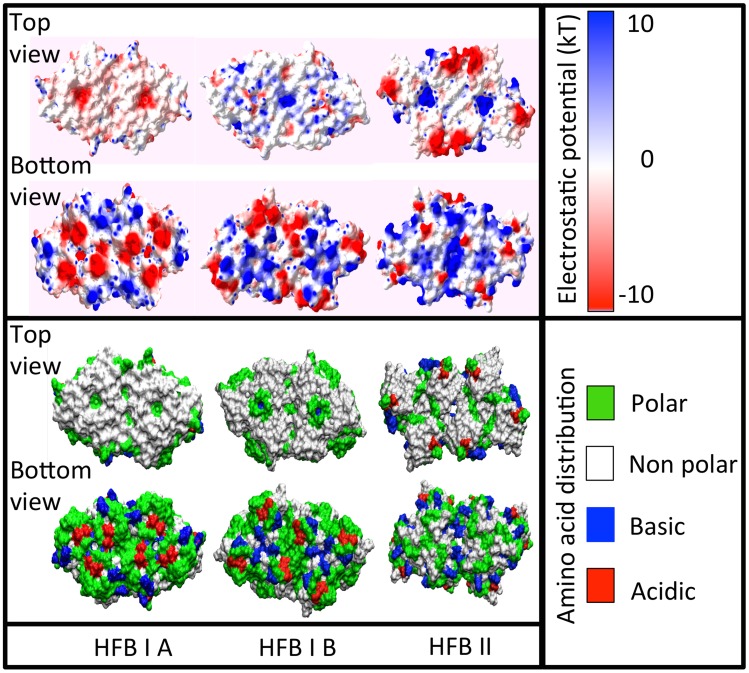
Rendering of the three hexamer structures showing top and bottom views, with both electrostatic potential and the amino acid distribution shown.

While the raw result of the protein-protein docking produced a structure that did not have exact P6 symmetry, we were, however, able to demonstrate that with minor adjustments to this structure, well within the accuracy of the docking calculation, a structure with exact P6 symmetry could be obtained. We iterated a genetic algorithm with energy minimisation to impose P6 symmetry. Disregarding the internal structure of the proteins, the protein arrangement within P6 symmetry is described by 6 parameters; the three Euler angles of the proteins in the trimer relative to the radial axis from the trimer center, the distance of proteins from their trimer center, the distance between the two trimers, and the rotational angle of the two trimers. The value of these six parameters closest to the docked structure was determined through direct geometric calculation, and the positions of the proteins were adjusted to the position conforming to the P6 symmetry. As a result of variances in the docked internal structures of the proteins, the resulting structure contained some clashes. These were resolved through alternately selecting new values for the six system parameters through a genetic algorithm and performing standard energy minimisation on the result. This process was repeated until the result converged. The resulting (.pdb) structures are found in the file “Dataset_S1.zip” in the Supporting Information (Dataset S1). Both the electrostatic potential, and amino acid distribution of the three hexamers are shown in Fig. 5. We see, as expected, that the air interface surface is both electrostatically neutral and non-polar.

It must be pointed out that we have so far only imposed the symmetry of the lattice and only taken into account the result from Langmuir film experiments [11], to the extent of the symmetry of the structure. Our results for the lattice parameter are in rough agreement with previously published experimental results for HFBI and slightly larger for HFBII: our hexagonal lattice has *a* = *b* = 54.7 Å for structure *α* and 57.1 Å for structure *β*, and for HFBII, *a* = *b* = 64.5 Å, in comparison to experimental results of Kisko *et al.* [11] of 55 and 56 Å respectively at zero surface pressure and 54 and 55 Å respectively under pressure. Maintaining the symmetry of the unit cell, we compressed the distance between the two trimers that make up the six protein unit cell to match the experimental lattice under pressure and were able to re-minimize the structure with only a small enthalpy gain and no significant unresolved clashes. Since our docking calculation involved no restructuring of the protein, it is to be expected that our results for the lattice parameter will err on the side of being too large; some minor reorientation of the loops in contact is to be expected. We can see that this is the cause of the result for HFBII showing more discrepancy with the experimental result than the case of HFBI: for HFBII there is a loop protruding into the contact region which can be easily restructured. The fact that our structures re-minimize perfectly when compressed to the experimentally determined structure supports this.

### Electron cryo-microscopy of two-dimensional crystals formed in water with six proteins in the unit cell and P3 symmetry

**Figure 6:**
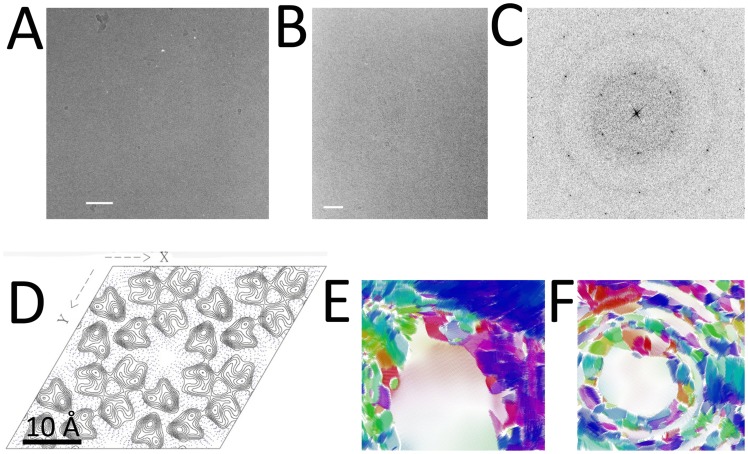
Results from electron cryomicroscopy: A) an unstained electron cryomicrograph of a HFBII film in water. Crystalline areas are slightly darker than non-crystalline areas. B) an unstained electron cryomicrograph of a HFBI film in water. Crystalline areas are slightly darker than non-crystalline areas. Scale bar is 100 nm in A and B. C) An inverted intensity image of the fast Fourier transform (FFT) of a selected HFBII area of the electron micrograph shown in A. D) A 2D projection map calculated from the HFBII image shown in A showing P3 symmetry, scale bar: 10 Å. Vector plots for distortion of HFBI and HFBII 2D crystals calculated during unbending showing high mosaicity in E) and F) respectively. Unit cell locations with vectors associated with higher noise are marked by coloured regions. Images were generated with the 2dx software package [47]. F) Is calculated from the HFBII image shown in A).

We conducted electron cryo-microscopy studies of HFBI and HFBII films forming ordered two-dimensional crystals in water in parallel with the protein-protein docking. A surface film was seen to form on 3*µ*l drops of both aqueous HFBI and HFBII solutions sitting on holey carbon coated copper grids. The HFBII film was formed using a protein concentration that was a hundredfold greater than the case for HFBI. The film formed in seconds for HFBII, for HFBI the film required up to 10 minutes to form at room temperature. The drops were carefully blotted with filter paper and vitrified for imaging at zero-tilt by electron cryo-microscopy. In the resulting micrographs, we found arrays of HFBI and HFBII. The water layer contained multiple crystals, in a mosaic array, and some of these were sufficiently large and ordered for Fourier analysis. When these were analysed by Fourier methods, it was obvious that some of the arrays were actually two-dimensional crystals which diffracted. Although the micrographs of both the HFBI and HFBII films contained more than one ordered areas, representing regions of single 2D crystals (Fig. 6). Several hundred images of both HFBI and HFBII were scanned for detectable crystals, and 16 micrographs of HFBI and 12 micrographs of HFBII containing crystals were found. For the HFBII preparations we were able to find several processable images with good statistics giving the same solution, however for HFBI, while the images gave similar diffraction patterns, and lattice parameters, the data were not to high resolution, and thus although we could show the same film formation and mosaicity, we were unable to obtain any processable images. Three images of HFBII were processed (Fig. 6 and Dataset S1 and S2 in Supporting Information). In agreement with x-ray scattering experiments the lattices were hexagonal with *α* = *β* = 56 Å, γ = 120° (see supplementary tables S1 and S2). Analysis of the phases of the Fourier patterns suggests a structure composed of a hexamer of proteins with density and lattice parameters in agreement with the docking results, but with P3 rather than P6 symmetry (Fig. 6). Possible reasons why the result shows P3 rather than P6 symmetry are discussed below.

### Role of a metastable low density P3 structure in accelerating film formation rate demonstrated through Monte Carlo simulation

**Figure 7:**
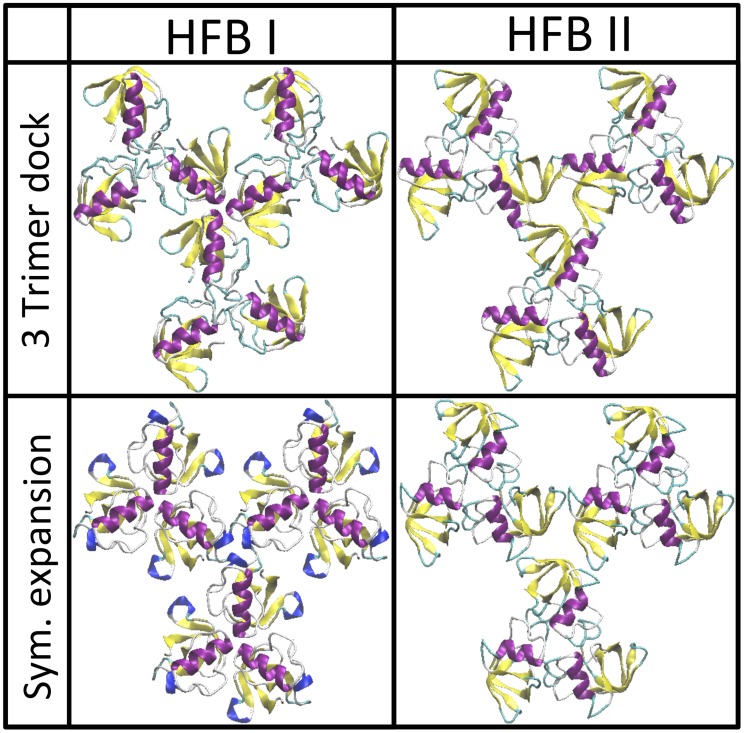
Results demonstrating verification of the 3 protein unit cell structure with P3 symmetry, protein-protein docking matches symmetric creation from the 5 protein structure. The results were successful, docking both the A and B trimers for both HFBI and HFBII.

Returning to the pentamer protein structure shown in Fig. 3, while this structure cannot be expanded into a structure with a 5 protein unit cell (Fig. 1 c) the structure can, however, be expanded into a surface covering lattice in a different manner, as a hexagonal lattice with a unit cell comprised of three proteins with the two vertexes of the structure being trimers A and B, as shown in Fig. 4. In order to verify this we performed protein-protein docking of three trimer structures, and were able to successfully duplicate this result, as shown in Fig. 7. This structure cannot be the experimentally observed surface layer structure; neither the lattice size nor surface density match the experimental value. The lattice size is 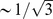 of the lattice size of both our computational and experimental results; the result is well outside the error bars for both results. The fact that this structure can be made is unlikely to be a coincidence, and a discussion of the relevance of this structure follows. The same symmetrization operation as performed for the structure with the six protein unit cell could be performed for this structure, and a continuous sheet of this structure could also be completed, as shown in Fig. 4 comparing the two structures. This structure belongs to the point symmetry group P3 [28].

The six protein structure found for HFBII, and the six protein structure *α* for HFBI are able to restructure from the P6 symmetric structure to the P3 symmetric structure through a simple rotation with very minor structural readjustment as shown in an animation, “Movie_S1.mpg”, included in the Supporting Information. There is no experimental evidence of this particular P3 structure existing as a stable state; the structure has a different, lower, lattice parameter than the P3 structure we have experimentally observed, and unlike the experimentally observed structure, has a three protein rather than six protein unit cell, however, this does not preclude links with this structure forming temporarily during the formation of the hydrophobic film. This P3 symmetry structure can be seen as a metastable structure. Proteins capable of forming 2D crystal structures with both P3 and P6 symmetries are not without precedent: the Annexin A5 protein forms both P6 and P3 structures on lipid bilayers [31], [32]. For this system, however, the P3 structure represents a more compact rather than expanded structure, that the system collapses to with increased lateral pressure.

**Figure 8:**
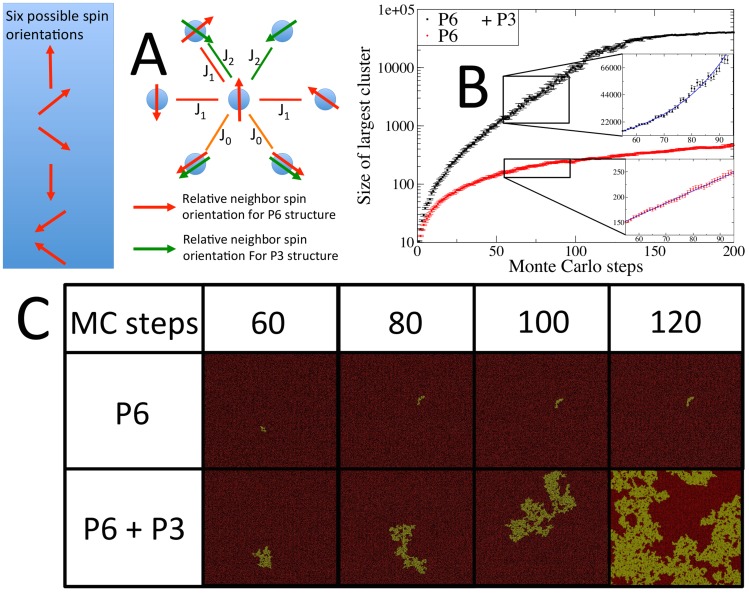
A) Schematic showing the construction of our spin model that allows for both P6 and P3 symmetry ordering on a triangular lattice as a simplified model of the hydrophobin surface. B) Plots of the largest cluster size vs. Monte Carlo time for spin model where both P3 and P6 symmetry are permitted, and one where only P6 is permitted. We see that the size of the largest cluster increases linearly for the system with only P6 symmetry and exponentially for the system where both the P3 and P6 symmetries are permitted. C) Visualization of both systems where only P6 ordering is allowed and where both P6 and P3 ordering are allowed, at 60, 80, 100, and 120 Monte Carlo steps. Protein positions on the triangular lattice are red and the largest cluster is shown in yellow. The much faster exponential, as opposed to linear, growth in the cluster size for the system with both P6 and P3 ordering can clearly be seen; the largest cluster percolates the 40×40 unit cell system at 120 Monte Carlo steps.

In order to explore the role of this possible ordering in the long range properties of the hydrophobin film, we have constructed a spin model that allows for both P6 and P3 local ordering, or only P6 ordering. The model involves spins in six possible orientations, each orientation a 60° clockwise rotation from the previous, on a hexagonal (triangular) lattice. A specific lattice site can either contain a spin or be vacant, representing the presence of absence of a hydrophobin at the surface. Each spin has six neighbour interactions, dictated by the angle between the spin and the spin at the given neighboring site. In order to incorporate both possible symmetries a minimum unit cell of the triangular lattice was 14×14 sites. The two possible symmetries are selected through the allowed neighbour interactions, as described in Fig. 8a.

The model involves three different interactions: between proteins (spins) in the same trimer (−*J*_0_), between proteins in neighboring trimers in the P6 structure (−*J*_1_), and between proteins in neighboring trimers in the low density P3 structure (−*J*_2_). The relative values of *J*_0_, *J*_1_, and *J*_2_, that match the real hydrophobin film, are possible to obtain through force biased simulation of the all atom model. We, however, have not performed this here, the relevance of the specific values of of *J*_0_, *J*_1_, and *J*_2_ is to be discussed in a future publication. All near neighbour spin orientations apart from the specific symmetries allowed (either P6 + P3 or only P6) are forbidden (infinite energy). In this study we have performed Monte Carlo simulation on this model with *J*_0_ = 5/*k_b_*, *J*_1_ = 2/*k_b_* and *J*_2_ = 1/*k_b_* for both P6 and P3 symmetry structures permitted, and *J*_0_ = 5/*k_b_* and *J*_1_ = 2/*k_b_* for only the P6 symmetry structure permitted. The specific values of *J*_0_, *J*_1_ and *J*_2_ were decided based on the following reasoning: the trimer interaction is seen to be far more stable than the other interactions and the P6 interaction has significantly greater protein contact area, in comparison to the P3 interaction. In addition to the spin – spin interactions we included an energetic benefit to the presence of a protein at the surface, to simulate the effect of the reduction in surface energy due to the presence of an amphiphilic hydrophobin. This energy was chosen to be *J_surf_* = −10/*k_b_*, to be significantly greater than any spin-spin interaction as indicative of the dominance of the amphiphilic nature of the hydrophobins. Similar spin models have been used in the past to model biological self assembly [32], [33].

Since the specific question we intend to answer is the role of the P3 metastable symmetry in the formation of the hydrophobin film, we designed the Monte Carlo algorithm that we performed on this specific interaction set (Hamiltonian) with the Monte Carlo steps designed in a fashion that mimics the possible motions of the individual hydrophobin proteins in the formation of the film. We thus allowed the following trial moves: 1) a spin appearing or disappearing on the lattice - corresponding to a hydrophobin rising to the surface or disappearing down off the surface 2) a spin hopping to a neighboring empty lattice site and/or rotating 60° and 3) a spin trimer moving one lattice site and/or rotating 60° together. Trial move type 2) corresponds to a single hydrophobin protein diffusing across the surface, and trial move 3) corresponds to tightly bound trimers diffusing in the same fashion as individual monomers.

Since the length scale of the objects/interactions being simulated is too large for temperature effects to have real physical meaning, the temperature was chosen to realise an ideal balance between system fluidity and the degree of ordering. The important property we measured was the size of the largest cluster of interacting spins as a function of Monte Carlo time. The rate of the growth of this is directly indicative of the rate at which the hydrophobin film forms an elastic network; the elastic properties of the hydrophobin film depend on the network of proteins connected by attractive interactions percolating the surface. In order to probe the role of the aforementioned metastable P3 structure, we monitored the rate of growth of the largest cluster in two separate models: 1) a model where both P6 and P3 symmetries are permitted, but the P6 structure is in the ground state and 2) a model where only the P6 structure is permitted.

Our Monte Carlo simulation result was striking: The rate at which contiguous regions of connected proteins grow increases dramatically when the P3 metastable interaction is allowed. We show this result in the plot in Fig. 8b, and a visualisation of this result is shown in Fig. 8c. In both cases there is an initial phase of increasing growth rate as the surface is being filled in, followed by a steady state region, where the growth is both independent of the initial conditions and finite size effects, thus our effective window on the real infinite system. When the cluster size reaches the scale of the system size then the rate levels off, and this can be seen as a finite size effect not relevant to gaining insight into the real system, however, this can be seen as analogous to growth up to a saturation level that occurs on a much larger length scale in an experimental system. We see that when the P6 symmetry alone is permitted the growth of the surface area of the largest cluster is linear in Monte Carlo time. When the P3 symmetry interaction is allowed the rate at which the largest cluster size grows not only increases faster in the steady state region, but the increase is exponential rather than linear. We then simulated the effect of unsaturated hydrophobin density by adding a certain probability, that when a space at the surface is to be filled by a hydrophobin, there is no hydrophobin present to fill it. With this probability set to 50%, we found the exponential cluster growth did not occur (data not shown). An interpretation of the reason for this, and thus the role of the P3 interaction in the formation of the hydrophobin film, is described in the discussion section.

### Molecular dynamics simulation demonstrates stable hexamer structure

We performed molecular dynamics simulations for 200 ns using the three hexamer structures determined from the docking calculation as starting configurations. The simulations were performed with the hydrophobins at the air-water interface and constant volume conditions with the unit cell set to the experimentally determined hexagonal lattice unit cell of length 56 Å. From the RMSD and hydrogen bond network formation we found the structure to equilibrate after 100 ns. The hexamer structure with approximate P6 symmetry maintained its integrity throughout the simulation. H-bond and salt bridge analysis has been performed (Dataset S3 in Supporting Information). Of particular note is the dominant salt bridge in the HFBI *β* structure between LYS 32 and ASP 30. This suggests that this is the more stable structure in comparison to the HFBI *α* structure, however a set of H-bonds and salt bridges is found for both structures so the results are inconclusive. These results could be used in a future mutagenesis study to test which of these structures is correct.

## Discussion

Through both protein-protein docking and electron microscopy analysis of vitrified film experiments we have determined that the structure of both HFBI and HFBII are composed of a six protein unit cell. The docking results, though not precisely P6, indicate a structure with P6 symmetry. The electron cryo-microscopy results, however show a structure with a six protein unit cell, but with P3 symmetry. Our result from the two-dimensional crystals gave lattice parameters of 56 Å for both systems. The lattice parameters found for HFBII crystals in cryoEM were similar to those reported in previous experiments [9]–[11]. The thin self-assembling films of HFBI and HFBII formed spontaneously in pure water, producing fragile crystals which are likely to be the ground state of the protein layer at the air-water interface in a thin film. However, the mosaicity within the films, and potentially, the lower order P3 symmetry observed compared to the P6 symmetry from simulations, may have been induced by the blotting procedure prior to vitrification, or by beam induced movement [34]. Another explanation could be the differences in entropy experienced within a thin water film, compared to bulk water. Recent findings from experiments [16] and simulations [15] with the class I hydrophobin EAS, that forms rodlets, has shown that the structures and conformational entropy of the class I hydrophobin EAS are substantially different when the protein is assembling in the air-water interface or is in bulk water. Finally, as we only have projection data for the crystals, we can not rule out the possibility that the crystals actually contain two layers of protein, one at each air-water interface, and thus an inherently different structure to that in the simulation where a single protein layer was assumed.

The process of the formation of the surface film, while an interesting question, is beyond the scope of this work, and the study of this is a possible future project. We can, however, state that there is no direct link between the tetramer found in the crystal structure and the film structure, since the contact points in the crystal structure are the hydrophobic surfaces at the air-water interface in the film.

From our protein-protein docking results we were able to independently obtain docked structures with P6 symmetry. For the HFBII system these showed a lattice parameter slightly larger than both the existing experimental result [9]–[11] and the new result we found. The system could, however, easily be compressed and re-minimized to the experimental lattice parameter. In addition to the near P6 structure our protein-protein docking results also yielded a lower surface density structure with near P3 symmetry but a lattice size that is not in agreement with the experimental results, thus a structure not experimentally observed. For the sake of clarity, it must be stressed that there is no relationship between this low density P3 structure, and the P3 structure found in the cryo-EM results, that has approximately the same lattice parameter and density of the P6 structure found in the docking results. We then explored the possible relevance of this low density P3 structure in the formation of the hydrophobin film using a Monte Carlo model.

Our docking results yielded two structures with near P6 symmetry for the hydrophobin HFBI, that we labelled *α* and *β*, and one structure for HFBII. Molecular dynamics (MD) simulations performed for 200 ns on all three structures showed equilibration to a stable structure at 100 ns. From our MD simulation we were able to identify a key set of H-bonds and salt bridges that could form between the proteins in each structure. Targeted mutagenesis of these key residues, coupled with studies of film formation could be used to distinguish whether the *α* or *β* structure for HFBI is correct. For both the HFBI *α* structure and the HFBII structure the transformation between the P6 structure and the metastable low density P3 structure, that we found for both proteins, could be achieved through a simple rotation, shown in the animation provided in Supporting Information. In the Monte Carlo analysis, to be discussed next, we assumed the P6 and low density P3 structures to be linked in this fashion.

We have performed a Monte Carlo simulation of a spin model constructed to investigate the effect of allowing for the P3 lattice on the formation of the hydrophobin film. When the P6 symmetry alone is permitted, the growth of the surface area of the largest connected cluster is linear in Monte Carlo time. When the P3 symmetry interaction is allowed, the rate of increase is exponential rather than linear. When the P6 lattice alone is permitted the system has 7 possible ordered structures, corresponding to the unit cell shown in Fig. 8a centered around each of the seven sub-lattices. The model thus roughly maps to a spin model known as a “two dimensional seven state Potts model”, belonging to the class of “two dimensional Q-state Potts models”. It has been shown that for this class of models the domain size scales roughly as 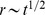 [35], where *r* is the radius of the region assumed to be circular and *t* is Monte Carlo time. This corresponds to linear expansion of the surface area of the domains in Monte Carlo time, exactly as we observe in our simulation. When the P3 symmetry interaction is allowed, P3 symmetry links can form between neighboring domains along the domain boundaries. Each time such a link is formed the area of the connected cluster doubles. Since the probability of such a link forming along the domain boundaries is constant, the rate at which this event occurs, thus doubling domain size, is also constant. The result is the domain size doubling at a constant rate: exponential growth. We additionally found that when we limit the availability of hydrophobin proteins to fill new holes at the surface, thus simulating below saturation concentration of hydrophobin proteins, the exponential growth in the domain size no longer occurs. This could explain the observed difference in the time for film formation between the two experiments for HFBI and HFBII, 10 minutes and seconds respectively, since the HFBII films were formed at a hundred fold higher concentration than the HFBI films.

We thus see evidence that the protein is specifically evolved to have both the P6 and P3 interactions with the P6 interaction dominant, but with the P3 interaction playing an important role; greatly accelerating the rate at which a new percolating network is formed within the hydrophobin film when it is subject to perturbation. This may contribute to the enhanced elasticity of this layer, and act as an additional mechanism to the mechanism of folding resulting in multilayers on the surface [36].

We have thus found an entirely new mechanism in the self organisation of biological structure which could play a role in a wide range of biological phenomena where effective 2D crystals of proteins are laid down on surfaces, including, for example, complement activation [37] in the human bloodstream, where we see a similar extremely fast growth of ordered protein surfaces. Taking an even broader perspective, this mechanism can be applied in developing biomimetics to construct amphiphilic nanoparticles with tuned interactions able to order in both the extended P3 and compressed P6 symmetry group structures at the water surface, possibly imparting novel properties to the surface as a result of this.

### Supporting Information

Along with this text there are a set of atomic model files included in PDB format. The .zip file “Dataset_S1” contains eight .pdb files. For each of the three hexamer structures (*α* (A) and *β* (B) for HFBI and HFBII) there is one copy of the raw docking result with suffix “_raw” and one copy of the six protein structure squeezed to the experimental lattice parameters under pressure of 54 and 55 Å respectively (Kisko *et al.* [11]) with suffix “_exfit”. For all cases the lattice vectors are along the x axis and rotated 120° counterclockwise from the x axis. Thus for HFBI with the lattice distance constrained to match the experimental system the lattice vectors are a = 54.00,0.0,0.0 b = −27,46.765,0.0 and for HFBI *α* structure with raw docking fit a = 54.723,0.0,0.0 b = −27.362,47.392,0.0, HFBI *β* is a = 57.113,0.0,0.0 b = −28.557,49.461,0.0. For HFBII the lattice vectors are a = 64.537,0.0,0.0 b = −32.269,55.891,0.0 and when constrained to fit the experimental lattice size a = 55.0,0.0,0.0 b = −27.5,47.631,0.0. The two structures of the metastable low density P3 structure with the three protein unit cell are also included. Both of these structures are aligned in the xy plane with lattice axis along x axis, like the six protein structures, and include a triangle of three unit cells. The Supplementary tables “Table_S1.pdf”, “Table_S2.pdf” and “Table_S3.pdf”, are also included, with S1 and S2 concerning details of the electron cryo-microscopy results, and S3 concerning results from the molecular dynamics simulation. The animation “Movie_S1.mpg” demonstrates the simple transition between the P6 and extended P3 2D crystal structures, described in the text.

## Materials and Methods

### Protein-protein docking

All protein docking studies were carried out using the MZ-dock package, developed by Pierce *et al.* [38]. MZ-dock uses a grid based approach for determination of the multimeric structure of the protein. In all cases resulting configurations that scored in the top 1% were then considered for further study, and a subset of these was selected based on criteria described below. Regarding the protein structures used in the docking, for HFBI we used chain b of structure 2FZ6 from the PDB database (resolution 1.67 Å). For HFBII, chain a of structure 1R2M was used (resolution 1.0 Å). In both cases the specific chain was chosen to be the chain with the longest defined protein structure.

As described above, the basic structural unit was deduced to be a trimer of three proteins, and docking of three proteins in this fashion was attempted, with the constraint on the docking algorithm that the major hydrophobic surface be blocked from docking, and all results where this surface is not oriented in the same direction for all three proteins as a flat surface were manually discarded.

Once protein trimers were determined, the larger scale structures, composed of many trimers (described in the results section) were determined by attempting docking of trimers to each other. For these docking attempts the trimer structures, discovered previously, were held fixed. The highest scoring structures were screened manually to remove all results where the major hydrophobic surface of the proteins did not form a flat structure.

### Molecular dynamics simulation

In order to further investigate the structure of the three determined hexamer structures (HFBI *α*, HFBI *β*, and HFBII) we performed 200 ns simulations of the structures determined through docking with the lattice size set to the experimentally determined value of 56 Å. Through periodic boundary conditions we were able to simulate a fully coated air-water interface through the simulation of the single hexamer with the hydrophobic surface exposed at the air-water interface. the three simulations were performed with a 1 fs time step and the simulation was carried out for 200 ns. We have followed the same methodology as used by Abigail *et al.* [39] for the simulation of the proteins at the air/water interface. We have used the Amber99 force field [40] with TIP3P water model within the Gromacs [41] software package to perform the molecular dynamics simulations at constant volume. The covalent bond lengths were preserved using the linear constraint solver (LINCS) algorithm [42]. All systems were simulated at constant volume and number of particles with the temperature controlled using the Nosé-Hoover thermostat [43], [44], with solvent and solute controlled independently. Lennard-Jones interactions were cut off at 1.0 nm, and for the electrostatic interactions the particle mesh Ewald method (PME) [45] was used. All simulations were carried out at ambient temperature (298 K).

### Electron microscopy of Langmuir film

The HFBI film was prepared by reconstituting dry powder in fresh milliQ water (pH 7) to a concentration of 10 mg/ml, sonicating for 30 seconds, and then diluting, to reach a concentration of 100 *µ*g/ml, followed by a second sonication. A 30*µ*l drop at that dilution was incubated in a closed petri dish for one hour at room temperature and atmospheric pressure. A visible film formed on top of the drop. The film was picked up with a freshly glow-discharged Quantifoil R2/2 (Quantifoil MicroTools GmbH, Germany) grid, then blotted and vitrified as described previously. The HFBII film was prepared by reconstituting dry powder in milliQ water to a concentration of 10 mg/ml and sonicating for 30 seconds. The HFBII system was not further diluted, thus the film was prepared in conditions of hundredfold greater protein concentration than the case for the HFBI film. After aliquoting 3*µ*l onto freshly glow-discharged Quantifoil R2/2 (Quantifoil MicroTools GmbH, Germany) grids at room temperature and atmospheric pressure, the drop was blotted from the front of the grid within a few seconds and then vitrified as described previously [46]. The grids were held in a GATAN 626 cryoholder maintained at −180°C in an FEI Tecnai F20 microscope (EM Unit, Institute of Biotechnology, University of Helsinki) operated at 200 kV. Images were recorded on Kodak SO163 film at a magnification of ×50,000 and the negatives were digitised using a Zeiss Photoscan TD scanner with a 7*µ*m step size. Micrographs were processed using the 2dx software package [47].

## Supporting Information

Dataset S1 A .zip file, “Dataset_S1.zip” containing files: “HFBI_A_raw.pdb” P6 hexamer structure *α* for HFBI raw fit, “HFBI_A_exfit.pdb” P6 hexamer structure *α* for HFBI fit to experimental lattice parameters, “HFBI_B_raw.pdb” P6 hexamer structure *β* for HFBI raw fit, “HFBI_B_exfit.pdb” P6 hexamer structure *β* for HFBI fit to experimental lattice parameters, “HFBII_raw.pdb” P6 hexamer structure for HFBII raw fit, “HFBII_exfit.pdb” P6 hexamer structure for HFBII fit to experimental lattice parameters, “HFBI_3_trimer.pdb” Low density P3 trimer structure for HFBI, “HFBII_3_trimer.pdb” Low density P3 trimer structure for HFBII.
(ZIP)

Movie S1 “Movie_S1.mpg” shows transformation between P6 and P3 structure.
(MPG)

Table S1 Image statistics for electron cryo-microscopy images of HFB II.
(PDF)

Table S2 Number of unique reflections and phase residuals in each resolution range.
(PDF)

Table S3 The H-bonds and salt bridges (highlighted in green) between proteins in both structures of HFBI and the structure for HFB II present for more than 20% of the trajectory. Note the dominant salt bridge between LYS32 and ASP30 in the HFB1 *β*.
(PDF)
