# Supplementary material for: Hydrophobin Film Structure for HFBI and HFBII and Mechanism for Accelerated Film Formation
Source: PLoS Comput Biol. 2014 Jul 31;10(7):e1003745. doi: 10.1371/journal.pcbi.1003745 (PMC4117420; doi:10.1371/journal.pcbi.1003745)
Supplement: Table S1 — Image statistics for electron cryo-microscopy images of HFB II. (PDF) [file pcbi.1003745.s003.pdf]

Supporting Information, Supplementary tables:

**Image statistics (projection structure)**	**Image 1**	**Image 2**	**Image 3**
Plane group symmetry	**p3**	**p3**	**p3**
Unit cell parameters	**a=b= 56.27 ± 0.06**<br>**γ= 120.01°**	**a=b= 56.28 ±0.15 Å**<br>**γ=120.23°**	**a=b= 55.92 ±0.37**<br>**γ= 120.04°**
Range of Defocus (Å)	**376645-38544**	**32985- 32210**	**36284-37924**
Total # of unique reflections to10 Å	**27**	**27**	**35**
Overall phase residual to cutoff at 10 Å	**15.291°**	**11.267 °**	**14.070 °**

Supplementary table S1: image statistics for electron cryo-microscopy images of HFB II.
