# Supplementary material for: Hydrophobin Film Structure for HFBI and HFBII and Mechanism for Accelerated Film Formation
Source: PLoS Comput Biol. 2014 Jul 31;10(7):e1003745. doi: 10.1371/journal.pcbi.1003745 (PMC4117420; doi:10.1371/journal.pcbi.1003745)
Supplement: Table S2 — Number of unique reflections and phase residuals in each resolution range. (PDF) [file pcbi.1003745.s004.pdf]

Supporting Information, Supplementary tables:

**Resolution range (Å) (Micrograph 1)**	**Number of unique reflections**	**Phase residual**
**1000.0-20.0**	**9**	**8.0°**
**20.0-14.1**	**9**	**19.9°**
**14.1- 11.5**	**4**	**17.4°**
**11.5-10.0**	**5**	**18.5°**

**Resolution range (Å) (Micrograph 2)**	**Number of unique reflections**	**Phase residual**
**1000.0-18.0**	**12**	**8.1°**
**18.0-12.7**	**5**	**0.6°**
**12.7- 10.4**	**8**	**14.5°**
**10.4-9.0**	**2**	**43.6°**

**Resolution range (Å) (Micrograph 3)**	**Number of unique reflections**	**Phase residual**
**1000.0-18.0**	**15**	**8.6°**
**18.0-12.7**	**8**	**13.3°**
**12.7- 10.4**	**12**	**21.4°**
**10.4 -9.0**	**0**	**0°**

Supplementary table S2: number of unique reflections and phase residuals in each resolution range.
