# Supplementary material for: Hydrophobin Film Structure for HFBI and HFBII and Mechanism for Accelerated Film Formation
Source: PLoS Comput Biol. 2014 Jul 31;10(7):e1003745. doi: 10.1371/journal.pcbi.1003745 (PMC4117420; doi:10.1371/journal.pcbi.1003745)
Supplement: Table S3 — The H-bonds and salt bridges (highlighted in green) between proteins in both structures of HFBI and the structure for HFB II present for more than 20% of the trajectory. Note the dominant salt bridge between LYS32 and ASP30 in the HFB1 β. (PDF) [file pcbi.1003745.s005.pdf]

Supporting Information, Supplementary tables:

HFBIα

Donor	Acceptor	Percentage
GLN70	GLN22	49.77
THR21	PRO10	33.62
THR71	ALA72	32.23
GLY25	GLN70	31.86
ASN4	ASN6	31.50
GLN22	PRO10	30.59
GLY3	LYS50	29.20
ASN37	ALA49	27.58
LYS32	ASN2	24.25
LEU12	GLN22	22.60
ASN2	THR51	22.44
GLY11	SER14	21.08

HFBIβ

Donor	Acceptor	Percentage
LYS32	ASP30	70.08
ASN6	ALA72	43.46
SER35	GLY52	31.99
LYS32	CYS18	29.62
THR21	VAL33	29.52
ASN37	SER35	23.58
GLN65	GLN65	28.25
ARG45	ASP40	20.28
GLN65	ALA63	20.18

HFBII

Donor	Acceptor	Percentage
THR5	PRO29	61.40
GLY6	ASN10	45.91
VAL2	PRO29	27.97
ALA1	ASP34	25.84
THR5	ASN10	25.59
GLY36	ASP34	25.40
THR5	PRO11	24.64
SER48	ASN17	24.51
ILE22	THR28	24.27
ALA1	VAL33	22.81

Supplementary Table S3. The H-bonds and salt bridges (highlighted in green) between proteins in both structures of HFB I and the structure for HFB II present for more than 20 % of the trajectory. Note the dominant salt bridge between LYS32 and ASP30 in the HFB1β
